# Usage Intention Toward an Interactive Smart Mirror Exercise Program Among Community-Dwelling Older Adults: An Application of the Decomposed Theory of Planned Behavior

**DOI:** 10.3390/healthcare14091120

**Published:** 2026-04-22

**Authors:** Yih-Ming Weng, Gia-Wei Chang, Meng-Siew Hii, Hsiu-Chun Chien, Jong-Long Guo

**Affiliations:** 1 College of Public Health, National Defense Medical University, Taipei 114201, Taiwan; wyming@mail.ndmutsgh.edu.tw; 2 Department of Health Promotion and Health Education, College of Education, National Taiwan Normal University, Taipei 10610, Taiwan; 80905005e@ntnu.edu.tw (G.-W.C.);; 3 Institute of Health Behaviors and Community Sciences, College of Public Health, National Taiwan University, Taipei 100233, Taiwan

**Keywords:** decomposed theory of planned behavior, exercise technology, health promotion, older adults, technology acceptance, advanced display technology

## Abstract

**Background/Objectives:** Sarcopenia and age-related muscle weakness pose significant global health challenges, highlighting the need for innovative and sustainable exercise interventions for older adults. This study developed and evaluated an Interactive Smart Mirror Exercise Program and investigated the factors associated with older adults’ usage intention toward the program based on the Decomposed Theory of Planned Behavior (DTPB). **Methods:** A cross-sectional survey was conducted with 92 community-dwelling older adults in northern Taiwan. Structural equation modeling was applied to test the proposed framework and examine the relationships among the study variables. **Results:** The results showed a satisfactory model fit (SRMR = 0.071). Attitude, subjective norms, and perceived behavioral control together explained 41.6% of the variance in usage intention. In addition, perceived usefulness, perceived compatibility, interpersonal influence, and self-efficacy were identified as factors significantly associated with usage intention, both directly and indirectly. **Conclusions:** These findings might support the applicability of the DTPB framework in explaining older adults’ usage intention toward technology-assisted exercise programs and provide insights for the design and implementation of digital exercise interventions in community settings.

## 1. Introduction

According to the latest statistics from the World Health Organization and the United Nations Population Fund, the global population is rapidly aging. The proportion of individuals aged 65 and above has increased from 5.5% in 1974 to 10.3% in 2024, with projections indicating it will exceed 16% by 2050 [1,2]. At that time, the global population will reach 9.8 billion, with the elderly population surpassing 1.5 billion, equivalent to one in every six individuals being an older adult. With advancing age, older adults are prone to muscle strength decline and physical deterioration, increasing the risk of falls and disability. Frailty has been demonstrated to be highly correlated with chronic diseases, hospitalization rates, and mortality [3]. Recent research indicates that technology-assisted exercise interventions should be included in guidelines to enhance physical activity in older people. For example, e-Health and digital exercise interventions have shown potential to increase the time spent on physical activity, energy expenditure in physical activity, and the number of walking steps [4] that might reduce frailty and delay functional decline. Therefore, how to engage older adults in physical activity to combat frailty in those with a lower level of technology acceptance becomes a critical issue [5].

Integrating technology-assisted smart mirrors with intuitive user interfaces may enable personalized feedback and health monitoring during exercise. Because these systems allow users to perform exercises directly in front of a display without wearable devices, they may also allow older adults to exercise in a more private and less intrusive manner [6]. In the context of smart mirror-based exercise systems, several system features—such as real-time feedback and interactive exercise activities—may be particularly relevant. For example, real-time motion feedback and virtual coaching functions may enhance older adults’ perceptions of usefulness and self-efficacy, while gamified exercise activities may increase perceived enjoyment and participation. In addition, the visibility of exercise participation in community settings may strengthen interpersonal influence and perceived social support. Therefore, the DTPB might provide a suitable framework for examining the factors associated with older adults’ usage intention toward smart mirror exercise systems [7].

Furthermore, although the benefits of interactive technology-based interventions over traditional exercise programs—particularly their ability to significantly improve dynamic balance among older adults—are widely acknowledged, users’ intention to use remains a critical challenge for their successful implementation. Integrating technology-assisted smart mirrors, and intuitive user interfaces not only enables personalized feedback and health monitoring, but also ensures privacy protection and non-invasiveness, thereby demonstrating both usability and feasibility. Such systems represent a highly promising digital solution for home-based elder care and institutional settings [7]. Nevertheless, the current literature primarily emphasizes physiological and cognitive outcomes, with comparatively limited exploration of older adults’ acceptance and use intention regarding interactive exercise programs. Hence, it is essential to adopt a behavioral-theoretical framework to elucidate the factors associated with older adults’ usage intention of technology-assisted interactive exercise programs.

With technological development, when exercise is combined with gamification elements, it not only increases the enjoyment of exercise but also enhances older adults’ usage intention [8]. The Interactive Smart Mirror Exercise Program (ISMEP) might incorporate game design, enabling older adults to exercise in a relaxed and pleasant environment, reducing stress and fatigue associated with exercise. Among these factors, real-time feedback and sense of achievement are considered important factors associated with older adults’ participation. Through advanced display technology, exercise games can provide real-time visual feedback, such as movement accuracy assessment and exercise completion calculation, while enhancing older adults’ confidence and sense of achievement through encouraging messages. Literature indicates that when older adults complete specific goals or challenges, the system can also provide reward mechanisms to further enhance engagement and motivation [9]. Gamification design significantly enhances older adults’ intention to use exercise programs through real-time feedback, sense of achievement, and personalized challenges [10].

This study presents ISMEP as an interactive and gamified program targeting the physical characteristics and psychological needs of older adults. To ensure safety, exercise program could adapt chair-based exercises, which can significantly reduce the risk of falls due to balance instability. Research has indicated that seated exercise is a safe and feasible alternative suitable for older adults, effectively maintaining physical function and mobility [11]. Additionally, exercise program incorporates real-time interactive feedback mechanisms, such as visual and auditory cues, enhancing older adults’ learning efficiency and exercise autonomy [12]—advantages that traditional one-way instruction does not possess. Prior literature also indicates that technology-assisted exercise program can improve older adults’ balance ability and movement performance through real-time feedback [13].

The Decomposed Theory of Planned Behavior (DTPB), proposed by Taylor and Todd, was developed based on Ajzen’s Theory of Planned Behavior (TPB) [14] and Davis’s Technology Acceptance Model (TAM) [15,16] to improve the explanatory power of traditional technology adoption models. In the original TPB framework, the use of single constructs (e.g., attitude and subjective norms) may limit explanatory ability because different underlying beliefs can offset one another [17]. To address this limitation, DTPB decomposes the main TPB constructs into multiple belief-based dimensions. Specifically, attitude is decomposed into perceived usefulness, perceived ease of use, and compatibility; subjective norms are decomposed into interpersonal influence; and perceived behavioral control is decomposed into self-efficacy, resource facilitating conditions, and technology facilitating conditions. This decomposition allows for a more detailed examination of factors associated with behavioral intention. Previous research on older adults’ use of robot-assisted board games demonstrated the applicability and strong explanatory power of the DTPB model, particularly highlighting the importance of perceived usefulness in shaping usage intention [18].

Guided by the DTPB framework, this study examined older adults’ usage intention toward the Interactive Smart Mirror Exercise Program (ISMEP). Attitude, subjective norms, and perceived behavioral control were proposed as antecedents of usage intention. Specifically, perceived ease of use, perceived usefulness, perceived enjoyment, and perceived compatibility were proposed as antecedents of attitude; interpersonal influence as an antecedent of subjective norms; and self-efficacy, resource-facilitating conditions, and technology-facilitating conditions as antecedents of perceived behavioral control [19].

## 2. Materials and Method

### 2.1. Study Design

The exercise demonstration routines were designed by faculty members and students from the Department of Physical Education at a university specializing in sports science for older adults. The instructor’s doctoral research specifically focused on exercise and safety in older adults, and the corresponding author is a gerontologist. Furthermore, to ensure operational safety, all exercises were demonstrated primarily in a seated position. The program consisted of 26 distinct movements, categorized into shoulder–neck–chest, upper limbs, and lower limbs. These demonstration movements were integrated into an advanced display system (smart mirror) (LiMART Co., Ltd., New Taipei City, Taiwan), enabling older adults to observe the movements and immediately follow along, while the system provided real-time feedback on their performance.

The structured exercise sequence followed the order of seven sessions: Introduction → Shoulder → Neck → Chest → Upper Limbs (Single Arm) → Upper Limbs (Both Arms) → Lower Limbs. Each movement was demonstrated via the high-definition display, after which participants performed the movement following the virtual coach’s guidance. The total duration of the exercise program depended on the participant’s movement speed. For participants who completed the movements more quickly, the program lasted approximately 15 min, whereas those who performed the movements more slowly required approximately 30 min.

To enhance engagement and enjoyment, four interactive exercise games—Stilt Walking, Shoulder Pole Balancing, Butterfly Catching, and Rowing—were incorporated. The four interactive games were specifically developed for this study. They were designed to enhance older adults’ upper- and lower-extremity mobility through traditional physical activities that are familiar to many older adults in Taiwan. For example, stilt walking simulates walking on stilts to improve lower-limb strength and balance; shoulder pole balancing simulates carrying items with a shoulder pole to increase shoulder range of motion; butterfly catching involves reaching to catch moving butterflies and is intended to improve body flexibility; and rowing is a virtual rowing activity designed to strengthen the upper limbs. These activities were incorporated because they may help older adults maintain or improve physical functions relevant to daily living activities. In addition, while playing these four games, participants were able to practice and reinforce the movements they had previously learned through the smart mirror system. Thus, the games served not only as a form of review, but also as a way to increase enjoyment and motivation during exercise. Each exercise game lasted about 3–5 min. All participants received equivalent exposure to the smart mirror exercise program, and that assistance provided by the research team was standardized across sessions.

These gamified components were designed to increase motivation and adherence among older adults. Participants could begin their fitness training simply by standing or sitting in front of the interactive smart mirror. Design of exercise program might simultaneously combine gamification and contextualization elements. This not only enhances program enjoyment but also strengthens intrinsic motivation through sense of achievement and gaming experience. Research has indicated that incorporating gamification and contextualization elements into exercise interventions for older adults can significantly enhance participation willingness and motivation while promoting physical and mental health [20,21].

**Figure 1:**
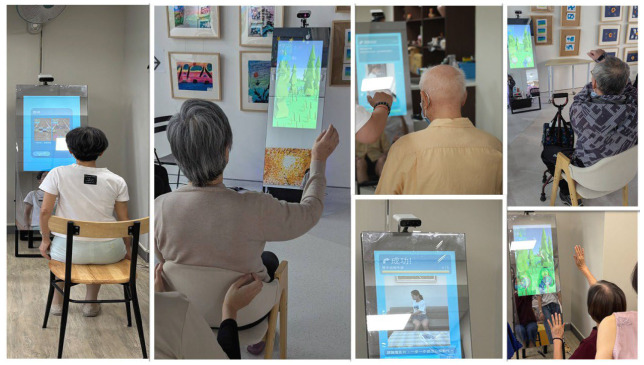
Interactive Smart Mirror Exercise Program.

During training sessions, the virtual coach displayed on the mirror not only demonstrated each movement but also evaluated the accuracy and completion level of participants’ actions. The inclusion of game-based elements and point-based task systems further enhanced older adults’ willingness to engage and continue participation in the exercise program. The design and operational processes of the program are illustrated in Figure 1.

### 2.2. Participants and Recruitment

The research team initially contacted more than ten community centers in Taipei City to explain the study objectives and procedures to the site administrators. Only two centers agreed to participate in. Among the centers that declined, the main reasons were scheduling incompatibility, the perception that existing exercise programs were already sufficient, and competing administrative or workload-related constraints. After obtaining institutional approval, recruitment posters were displayed at two centers, and orientation sessions were held to introduce the study details. Older adults who expressed interest were invited to join the study and were required to provide written informed consent prior to participation. In total, ninety-two community-dwelling older adults aged sixty-five years and above were recruited from the two participating community centers. Participants were eligible for inclusion if they were aged sixty-five or older, were active members of a community center, and possessed sufficient communication abilities without severe visual or auditory impairments that might interfere with participation. All participants were cognitively capable of understanding the study’s purpose and procedures, able to comply with the research instructions, and demonstrated a cooperative attitude toward the research process. Participation was entirely voluntary, and all individuals signed informed consent forms before enrollment.

### 2.3. Measurement

**Figure 2:**
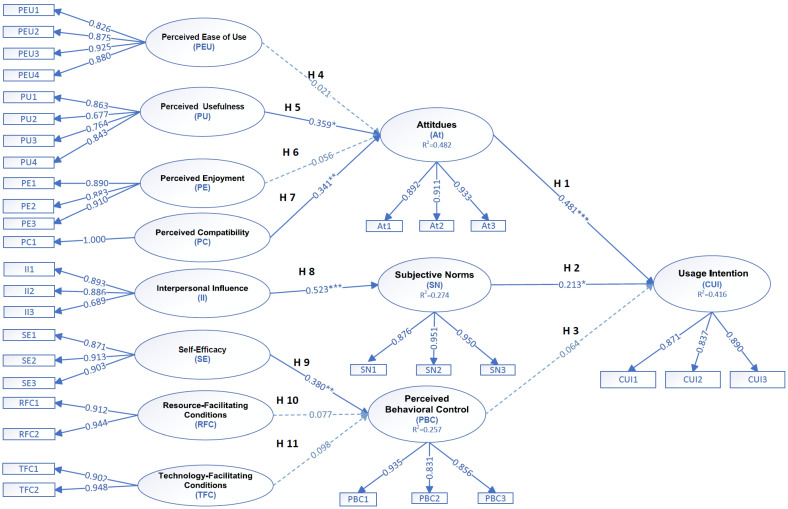
The overall structure of the proposed model. * *p* < 0.05, ** *p* < 0.01, *** *p* < 0.001.

The questionnaire based on the DTPB consisted of two major sections: sociodemographic variables and theoretical constructs derived from the DTPB framework. The demographic variables included participants’ gender, age, chronic health conditions, and interest in adopting the smart mirror as a learning tool in future use. The items were adapted from previously validated instruments developed in prior DTPB studies [18,22,23] and were further refined following expert validity review to form the final version of the research questionnaire. The DTPB-related instrument comprised three core constructs—attitude, subjective norms, and perceived behavioral control—along with their antecedent belief variables (e.g., perceived usefulness, perceived ease of use, resource facilitation, and self-efficacy), yielding a total of twelve variables. Each variable contained one to four items, resulting in thirty-four items in total as presented in Figure 2. All items were measured using a five-point Likert scale, where participants rated their level of agreement from 1 = strongly disagree to 5 = strongly agree. Higher scores indicated stronger positive evaluations or beliefs regarding the given construct. For example, a sample item under the attitude construct was: “I enjoy using the smart mirror to learn exercise movements.” A higher score on this item reflected a more favorable attitude toward participation in the program.

### 2.4. Statistical Analysis

The participants’ demographic characteristics were summarized using SPSS version 23.0 (IBM Corp., Armonk, NY, USA) and reported as frequencies and percentages. To test the proposed hypotheses, partial least-squares structural equation modeling (PLS-SEM) was performed with SmartPLS version 3.0 (SmartPLS GmbH, Oststeinbek, Germany). Compared with covariance-based structural equation modeling, PLS-SEM is considered more appropriate for research involving relatively small samples [24]. It is also well suited for evaluating theoretical models with an emphasis on prediction. For PLS-SEM, the recommended minimum sample size is generally 10 times the largest number of structural paths directed at any latent construct in the model [25]. In the present study, the maximum number of paths pointing to a single latent construct is four; therefore, according to this rule, the recommended minimum sample size would be 40. Since our study included 92 participants, the sample size exceeds this commonly used guideline. To ensure that the sample size was adequate for the structural model analysis, an a priori power analysis was conducted using G*Power 3.1.9.7 (Heinrich-Heine-Universität Düsseldorf, Düsseldorf, Germany). In the present PLS-SEM model, the maximum number of predictors for any endogenous construct was four. Assuming a medium effect size (f^2^ = 0.15), a significance level of α = 0.05, and a statistical power of 0.80, the analysis indicated that a minimum sample size of 85 participants was required. Since the present study included 92 participants, the sample size was considered sufficient for the statistical analysis [26,27].

The PLS-SEM procedure was carried out in two steps [28]. In the first step, the measurement model was evaluated to determine the reliability and validity of all constructs. In particular, convergent validity and discriminant validity of the latent constructs needed to be established. Convergent validity was assessed based on indicator loadings, Cronbach’s α, composite reliability (CR), and average variance extracted (AVE). Indicator loadings and Cronbach’s α values of 0.70 or above were regarded as acceptable. CR was used to assess internal consistency, with recommended values exceeding 0.70 [29]. AVE reflects the extent to which a latent construct explains the variance of its observed indicators, and values above 0.50 were considered acceptable. Discriminant validity was examined using the Fornell–Larcker criterion and heterotrait–monotrait (HTMT) ratio. According to the Fornell–Larcker criterion, the square root of the AVE for each construct should be greater than its correlations with other latent constructs [25]. HTMT values below 0.90 were considered evidence of adequate discriminant validity [30].

In the second step, the structural model was evaluated. Path coefficients were estimated to test the hypothesized relationships among constructs. Model fit was assessed using the standardized root-mean-square residual (SRMR), where lower values indicate better fit and values below 0.08 suggest acceptable model fit. SRMR has been widely recommended as a global approximate fit measure in PLS-SEM and is commonly reported in studies applying variance-based structural equation modeling [31]. Because global goodness-of-fit indices commonly used in covariance-based SEM (e.g., RMSEA, GFI) are not generally emphasized in PLS-SEM, SRMR was used as the primary model fit indicator in this study. The coefficient of determination (R^2^) was used to evaluate the proportion of explained variance. R^2^ values of 0.75, 0.50, and 0.25 are generally interpreted as indicating substantial, moderate, and weak predictive power, respectively [24].

### 2.5. Common Method Bias Assessment

Because all study variables were collected from the same respondents using a self-report questionnaire at a single time point, common method bias (CMB) was assessed both procedurally and statistically. Procedurally, participant anonymity was ensured and respondents were informed that there were no right or wrong answers, which may help reduce evaluation apprehension and method bias [32]. Statistically, Harman’s single-factor test was conducted, and the first factor accounted for 42.98% of the total variance, which is below the commonly suggested threshold of 50%. In addition, the full collinearity VIF approach was applied in the PLS-SEM model [33]. Most constructs showed VIF values below the recommended threshold of 3.3, although the resource-facilitation conditions (RFC) construct slightly exceeded this value. Overall, these results suggest that common method bias is unlikely to severely affect the results; however, the potential influence of method bias cannot be completely ruled out and should be considered when interpreting the findings.

### 2.6. Ethical Considerations

This study was approved by the Research Ethics Review Committee of the National Taiwan Normal University, No. 202505HM020. All participants were fully informed and provided their written informed consent before the study began.

## 3. Results

### 3.1. Participants Characteristics

A total of ninety-two valid questionnaires were collected, yielding a response rate of 100%. The 100% response rate was achieved because all 92 participants completed the questionnaire immediately after finishing the smart mirror exercise session. In addition, each questionnaire was reviewed individually by a member of the research team upon completion to confirm that no items were missing. As a result, all questionnaires were fully completed and included in the analysis, yielding a response rate of 100%. Among the participants, sixty-six were female (71.74%), and the largest age group was between sixty-five and seventy years (*n* = 34, 36.96%). Regarding health conditions, hypertension was the most commonly reported chronic illness (*n* = 30, 32.61%) (Table 1). Following their experience with the smart mirror, a substantial majority (*n* = 73, 79.35%) expressed interest in adopting the smart mirror as a learning tool, indicating potential acceptance of the technology for exercise-based applications.

In terms of descriptive statistics, the mean scores of the twelve DTPB-related variables ranged from 3.938 to 4.277, all exceeding the neutral midpoint of 3 on the five-point Likert scale. This suggests that participants generally held positive evaluations toward the ISMEP. Among the variables, technology-facilitating conditions recorded the highest mean score (Mean = 4.277), while interpersonal influence had the lowest mean score (Mean = 3.938). These results collectively demonstrate a favorable overall perception of the program among participants. Detailed results are presented in Table 2.

### 3.2. Measurement Model Assessment

Table 3 presents factor loadings, Cronbach’s α, composite reliability (CR) and average variance extracted (AVE) to support convergent validity. The factor loadings ranged from 0.677 to 1.000, Cronbach’s α values ranged from 0.768 to 0.916, CR values ranged from 0.785 to 0.937, and AVE values ranged from 0.624 to 0.861. The measurement model demonstrated acceptable convergent and discriminant validity for the multi-item constructs. Because perceived compatibility was measured with a single item, reliability indicators such as Cronbach’s alpha, composite reliability, and AVE could not be calculated for this construct. Therefore, the validity assessment primarily applies to the remaining multi-item constructs.

Furthermore, discriminant validity was evaluated using the Fornell–Larcker criterion and the heterotrait–monotrait (HTMT) ratio. As shown in Table 4, the square root of the AVE for each construct was greater than its correlations with other latent constructs, and all HTMT values were below the 0.90 threshold, confirming that discriminant validity was well-established.

### 3.3. Structural Model Assessment

A structural equation modeling (SEM) analysis was conducted to assess the overall adequacy and goodness of fit of the proposed model. The Standardized Root Mean Square Residual (SRMR) value was 0.071, which is below the conventional threshold of 0.08, indicating a satisfactory and well-calibrated model fit. Based on the results, four antecedent variables—perceived ease of use, perceived usefulness, perceived enjoyment, and perceived compatibility—collectively explained 48.2% of the variance in attitude toward use. The construct of interpersonal influence accounted for 27.4% of the variance in subjective norms, whereas self-efficacy, resource facilitation, and technological support conditions jointly explained 25.7% of the variance in perceived behavioral control. Finally, attitude, subjective norms, and perceived behavioral control together explained 41.6% of the variance in behavioral intention to continue using the ISMEP. Among the hypothesized paths, six were statistically significant, reflecting theoretically coherent predictive relationships among the constructs.

Figure 2 illustrates the examination of eleven hypothesized paths, analyzing how the various DTPB-related variables are associated with attitude, subjective norms, perceived behavioral control, and usage intention. The analysis revealed multiple statistically significant and positive relationships. The first significant path (H1) indicated that attitude exerted a positive effect on usage intention (β = 0.481, t = 4.875, *p* < 0.001). This finding suggests that individuals with more favorable attitudes toward the program were considerably more likely to maintain their intention to continue using it. Notably, attitude (β = 0.481) emerged as the most significant associated factor of behavioral intention among all significant paths, emphasizing its central position within the behavioral intention framework. The second path (H2) also demonstrated that subjective norms had a significant positive association with usage intention (β = 0.213, t = 2.312, *p* = 0.021). This implies that perceived social endorsement or expectations from significant others meaningfully reinforce individuals’ intention to use the system. The fifth path (H5) revealed that perceived usefulness was notably and positively associated with attitude (β = 0.359, t = 2.570, *p* = 0.010). This result supports the premise that individuals who perceive a program as beneficial or functionally valuable tend to develop more favorable attitudes toward its use. The seventh path (H7) showed that perceived compatibility was positively associated with attitude (β = 0.341, t = 2.789, *p* = 0.005). This finding indicates that when the system aligns with users’ personal beliefs, routines, or lifestyle, their attitudinal evaluations become increasingly positive. The eighth path (H8) demonstrated that interpersonal influence was highly and significantly associated with subjective norms (β = 0.523, t = 6.431, *p* < 0.001). This relationship exhibited the largest standardized coefficient among all significant paths, underscoring the salient role of social interaction in shaping perceived normative pressure and behavioral expectations. Finally, the ninth path (H9) showed that self-efficacy exerted a significant positive effect on perceived behavioral control (β = 0.380, t = 2.610, *p* = 0.009). This suggests that individuals who view themselves as capable and confident in performing the behavior perceive greater control over their engagement with the program. Six of the eleven hypothesized relationships—H1, H2, H5, H7, H8, and H9—were empirically supported, indicating that attitude, subjective norms, perceived usefulness, perceived compatibility, interpersonal influence, and self-efficacy are saliently associated with behavioral intention and related constructs. Detailed statistical outcomes are reported in Table 5.

## 4. Discussion

This study applied the Decomposed Theory of Planned Behavior (DTPB) to examine the determinants influencing older adults’ usage intention with an interactive smart mirror exercise program. The structural model demonstrated an acceptable fit, with attitude emerging as the most substantive determinant of behavioral intention. This finding aligns with prior research highlighting that older adults’ participation in digital health or technology-assisted exercise programs is tied to perceived personal relevance of the technology [34]. Attitude encapsulates both perceived enjoyment and compatibility, echoing the affective component of behavioral intention widely observed in technology adoption studies.

Consistent with previous findings, perceived usefulness and compatibility demonstrated are associated with positive attitudes toward system use, indicating that older adults value digital technologies when they are functionally beneficial, align with prior finding. The importance of perceived enjoyment identified in this study also corroborates recent finding suggesting that playfulness and engagement are crucial facilitators of adherence in technology-assisted exercise interventions [35]. Furthermore, our finding also supports the growing evidence that virtual coach promoting positive affect and usability can strengthen technology usage intention among community-dwelling older adults [36].

Social influences were also pivotally associated with older adults’ engagement with the smart mirror exercise program. Subjective norms—capturing perceived expectations from partner, family members, and relatives/friends—were found to be significantly associated with behavioral intention. This finding is consistent with prior studies indicating that social endorsement and normative pressure are salient motivators for older adults’ participation in digital health technologies [37]. Social connectedness, as facilitated by interactive technologies, has been identified as an essential factor promoting technology acceptance and reducing perceived isolation among older adults [38]. Future research could further investigate whether different sources of social influence (e.g., partners, family members, peers, or healthcare providers) exert distinct effects on older adults’ intention to use smart mirror exercise systems. Understanding these differences may help develop more targeted strategies to promote technology-assisted exercise among older adults.

Previous studies have suggested that exercise self-efficacy plays a vital role in promoting physical activity among older adults [39]. Exercise self-efficacy has also been shown to predict subsequent exercise behavior in older adults [40]. At the same time, participation in exercise interventions may strengthen individuals’ self-efficacy over time as they gain mastery experiences and perceive improvements in their physical abilities [39,40]. In addition, studies on technology-based exercise and exergames have indicated that older adults’ perceptions of technology for health promotion may become more positive after actual use, and enjoyable exergame experiences may further support confidence, engagement, and perceived competence [41,42]. Similar observations have also been reported in recent empirical investigations on digital health readiness, suggesting that technological self-efficacy mediates the relationship between perceived usability and sustained adoption of smart healthcare devices among older users [43]. Moreover, ensuring resource accessibility and facilitating conditions—such as intuitive interfaces and clear guidance—can further reinforce this confidence, as noted in prior behavioral design studies [44]. These findings emphasize that enhancing users’ sense of competence and minimizing technological barriers are crucial for sustaining participation in technology-assisted exercise interventions.

### 4.1. Theoretical Implications

The present findings extend the applicability of the Decomposed Theory of Planned Behavior to the domain of digital exercise technologies for older adults. Consistent with prior research, the present model demonstrates that perceived usefulness, compatibility are associated with attitudes toward usage intention with exercise technologies. The findings offer a more refined account of behavioral intention among older adults, highlighting that technology usage is associated with the interplay of both cognitive appraisals and affective evaluations rather than with a single evaluative pathway.

Moreover, the findings corroborate previous theoretical analyses demonstrating that DTPB offers an integrative framework for explaining behavioral intention in health-related technology adoption across varying levels of digital literacy. By applying DTPB to a smart mirror-based exercise program, this study reinforces the model’s theoretical suitability for investigating technology-mediated exercise behaviors. It also contributes to the growing body of literature integrating behavioral intention theories—such as TAM and TPB—to capture both instrumental and experiential factor of usage intention [45].

Moreover, the associations observed between self-efficacy and perceived behavioral control indicate that older adults’ participation with technology may involve considerations beyond cognitive appraisals alone, extending to perceptions of competency in the use process. Such observations are consistent with prior conceptual frameworks situated at the intersection of behavioral theory and human–technology interaction, which suggest that perceived control may play a role in linking self-efficacy with behavioral intention [22]. From this perspective, the present results offer tentative support for the applicability of the DTPB within interactive, technology-supported exercise program.

### 4.2. Practical Implications

The observed associations among attitude, subjective norms, and perceived behavioral control suggest that effective systems may need to address not only technical functionality but also users’ social and emotional considerations. This observation is consistent with the digital health literature, which indicates that factors such as usability, and perceived social support are associated with participation among older populations [46].

From a design standpoint, interactive platforms such as the smart mirror may benefit from an emphasis on user-centered adaptability. In particular, interfaces that combine intuitive operation with multimodal feedback could help accommodate the sensory and cognitive heterogeneity commonly observed among older adults. Prior studies have reported that features such as real-time performance feedback, visual guidance are positively associated with perceived enjoyment and usability, which in turn have been linked to system use [47]. In addition, incorporating functions related to virtual coaching or peer comparison may potentially reinforce subjective norms, as perceptions of social presence and validation have been associated with adherence in health-promoting physical activity programs [36].

In the study, the program was delivered through a chair-based exercise format that incorporated guidance from a virtual coach, motion-sensing-based feedback on participants’ movements, and on-site encouragement provided by a human caregiver. This combination of delivery strategies reflects an emphasis on accommodating participants’ physical capabilities while simultaneously offering instructional clarity and social support. Our findings suggest that supporting technological self-efficacy play a significant role in promoting the exercise intention. Strategies such as guided tutorials, adjustable difficulty levels, and community-based orientation sessions could help reduce apprehension toward unfamiliar technologies and facilitate greater confidence in system use. Consistent with prior finding, technology that incorporate accessibility-oriented features and context-aware adjustments appear more likely to be perceived as acceptable by older adults with diverse physical and cognitive capacities [48].

As digital health interventions are increasingly being applied in home-based and hybrid care settings, the compatibility of smart mirror programs with existing healthcare infrastructures may be one factor related to usage intention. Technology-supported tools may be regarded as complementary resources that could support the connection between clinical oversight and everyday wellness practices [49].

### 4.3. Limitations and Future Research Directions

This study has several limitations that should be considered when interpreting the findings. First, participants were recruited from only two community centers, although more than ten centers were initially contacted. In addition, eligibility criteria required participants to be active community members who could communicate adequately and comply with study procedures. Consequently, the sample may have more supportive management, more active community programs, or older adults who are relatively more open to participating in technology-related activities. Previous studies have shown that contextual and cultural factors may influence technology usage among older adults, suggesting that cross-cultural and multi-site studies are needed to better capture diversity in behavioral intentions [50]. Moreover, because the participants were primarily community-dwelling older adults with relatively high functional independence and cognitive ability, the results may not fully represent frail older adults or those with lower digital literacy. Limited technological confidence and digital literacy have been identified as important barriers to the adoption of digital health interventions, particularly among frail or socially isolated populations [51].

Second, this study employed a cross-sectional design, which limits causal inference among the constructs examined within the DTPB framework. The relationships identified in the structural model should therefore be interpreted as associations rather than causal effects. Longitudinal or experimental research designs would be helpful to examine how attitudes, social influences, and perceived behavioral control evolve over time with continued use of technology-assisted exercise systems [52].

Third, several methodological issues should be noted. The construct of perceived compatibility was measured using a single item, which may limit the assessment of internal consistency and construct reliability. In addition, because all variables were collected through self-report questionnaires at a single time point, common method bias cannot be completely ruled out. The resource-facilitation conditions construct may also have been associated with the provision of the smart mirror system, real-time audiovisual feedback, and assistance from the research team during the intervention. Although assistance was standardized across participants and all participants received equivalent exposure to the smart mirror exercise program, the presence of on-site human support may still have been associated with participants’ perceptions of the program and their reported usage intention. Furthermore, the absence of a control or comparison group means that the study cannot isolate the specific contribution of the smart mirror delivery mechanism from the possible enjoyment of the exercise content itself or the motivating effect of social support during the session. Participants completed the questionnaire immediately after the exercise session, and their responses may partly reflect a novelty or demonstration effect rather than real-world usage. Therefore, the findings should be interpreted as reflecting participants’ usage intention after initial exposure.

Future studies may further examine the role of smart mirror exercise systems through controlled or comparative designs, such as comparing smart mirror delivery with video-based or instructor-led exercise programs. In addition, future research could explore the integration of smart mirror systems into broader healthcare infrastructures, including electronic health records and remote monitoring platforms, which may support preventive care and chronic disease management in community or home settings [53]. Expanding future studies to include multi-site samples and broader participant groups, such as family members and caregivers, may also help clarify the role of social support in promoting participation with digital exercise technologies [54]. Finally, qualitative and mixed-methods approaches may provide deeper insights into older adults’ experiences, emotional responses, and contextual barriers when interacting with smart mirror exercise systems, thereby informing the design of more user-centered digital health interventions [55].

## 5. Conclusions

This study supports the applicability of the Decomposed Theory of Planned Behavior for examining older adults’ usage intention toward a smart mirror-based exercise program. The findings indicate that attitude toward the program showed the strongest association with usage intention, followed by subjective norms, whereas perceived behavioral control showed a weaker and non-significant relationship. Because perceived compatibility was measured using a single item, the psychometric robustness of this construct may be limited, and the observed relationship between perceived compatibility and attitude should therefore be interpreted with caution. Moreover, because this study employed a cross-sectional design, the relationships identified in the model should be interpreted as associations rather than causal effects.

Furthermore, the relative magnitude of the estimated path coefficients provides further insight into the relationships among the studied variables. Among the three determinants of usage intention, attitude showed the largest standardized coefficient (β = 0.481), suggesting that older adults’ overall evaluations of the smart mirror exercise program were more strongly associated with their reported intention to use the system than the other constructs examined in this study. Subjective norms demonstrated a smaller but statistically significant association with usage intention (β = 0.213), indicating that perceived expectations from partners, family members, or peers may also be related to older adults’ willingness to engage with the program. Furthermore, self-efficacy showed a significant association with perceived behavioral control (β = 0.380), suggesting that confidence in performing the exercises and interacting with the system may contribute to participants’ perceived ability to use the program. These findings suggest that both individual evaluations of the technology and social influences may be associated with older adults’ reported intention to use smart mirror-based exercise programs.

Beyond the theoretical implications, the findings also provide several practical insights for the design of smart mirror-based exercise systems for older adults. To enhance perceived usefulness and self-efficacy, developers may consider integrating a virtual coach that demonstrates movements clearly and provides step-by-step guidance during exercise sessions. Such virtual coaching content could be developed collaboratively by gerontology researchers, exercise specialists, long-term care professionals, and engineers, with movement demonstrations recorded by trained exercise science students to ensure clarity and safety for older users. In addition, incorporating motion-detection sensors that provide real-time feedback on movement accuracy may help users better understand their performance and reinforce confidence in completing the exercises.

Furthermore, incorporating gamified exercise activities that are culturally familiar to older adults, such as traditional physical games or activities commonly experienced during their youth, may increase enjoyment and participation with the system. For example, activities such as butterfly catching or rowing simulations can transform routine physical exercises into interactive experiences, potentially strengthening users’ perceived usefulness of the system while also promoting intention to exercise. Together, these design considerations may help improve the usability and acceptance of smart mirror-based exercise technologies for older adult populations.

## Figures and Tables

**Table 1:** Demographic results of study participants (*n* = 92).

Variables	Numbers (*n*)	Percentage (%)
Gender		
Male	26	28.26%
Female	66	71.74%
Age		
65–70 years	34	36.96%
71–75 years	32	34.78%
76–80 years	20	21.74%
≥81 years	6	6.52%
Disease		
Hypertension	30	32.61%
Diabetes Mellitus	13	14.13%
Heart Disease	7	7.61%
Cancer	1	1.09%
Liver Cirrhosis	1	1.09%
Others	12	13.04%
None	28	30.43%
Interest in adopting the Smart Mirror as a learning tool		
No	19	20.65%
Yes	73	79.35%

**Table 2:** Mean, standard deviation and score range of DTPB-related 12 Variable.

Variable	Item	Mean	Standard Deviation	Score Range
Attitudes	3	4.152	0.687	3–5
Subject Norms	3	3.957	0.657	3–5
Perceived Behavioral Control	3	4.236	0.581	3–5
Usage Intention	3	4.207	0.673	3–5
Perceived Ease of Use	4	4.185	0.524	3–5
Perceived Usefulness	4	4.193	0.541	3–5
Perceived Enjoyment	3	4.138	0.619	3–5
Perceived Compatibility	1	4.054	0.559	3–5
Interpersonal Influence	3	3.938	0.612	3–5
Self-Efficacy	3	4.199	0.509	3–5
Resource-Facilitating Conditions	2	4.065	0.574	3–5
Technology-Facilitating Conditions	2	4.277	0.603	3–5

**Table 3:** Convergent validity of the proposed measurement model.

Variables	Factor Loadings	Cronbach’s α	Composite Reliability	Average Variance Extracted
Attitudes (At)		0.899	0.910	0.832
At1	0.892			
At2	0.911			
At3	0.933			
Subject Norms (SN)		0.916	0.916	0.858
SN1	0.876			
SN2	0.951			
SN3	0.950			
Perceived Behavioral Control (PBC)		0.836	0.846	0.752
PBC1	0.885			
PBC2	0.831			
PBC3	0.886			
Usage Intention (UI)		0.898	0.937	0.832
CUI1	0.865			
CUI2	0.983			
CUI3	0.937			
Perceived Ease of Use (PEU)		0.900	0.906	0.769
PEU1	0.826			
PEU2	0.875			
PEU3	0.925			
PEU4	0.880			
Perceived Usefulness (PU)		0.796	0.810	0.624
PU1	0.863			
PU2	0.677			
PU3	0.764			
PU4	0.843			
Perceived Enjoyment (PE)		0.876	0.886	0.800
PE1	0.890			
PE2	0.883			
PE3	0.910			
Perceived Compatibility (PC)		-	-	-
PC1	1.000			
Interpersonal Influence (II)		0.768	0.785	0.690
II1	0.893			
II2	0.886			
II3	0.698			
Self-Efficacy (SE)		0.877	0.883	0.803
SE1	0.871			
SE2	0.913			
SE3	0.903			
Resource-Facilitating Conditions (RFC)		0.840	0.868	0.861
RFC1	0.912			
RFC2	0.944			
Technology-Facilitating Conditions (TFC)		0.835	0.890	0.855
TFC1	0.902			
TFC2	0.948			

**Table 4:** Results of the Fornell–Larcker criterion and HTMT ratio of correlations.

Variables	1.	2.	3.	4.	5.	6.	7.	8.	9.	10.	11.	12.
Fornell–Larcker criteriona												
1. Attitudes	0.912											
2. Subjective Norms	0.473	0.926										
3. Perceived Behavioral Control	0.432	0.435	0.867									
4. Usage Intention	0.609	0.468	0.364	0.867								
5. Perceived Ease of Use	0.516	0.467	0.480	0.547	0.877							
6. Perceived Usefulness	0.637	0.405	0.463	0.575	0.693	0.790						
7. Perceived Enjoyment	0.499	0.364	0.314	0.480	0.564	0.611	0.895					
8. Perceived Compatibility	0.630	0.422	0.376	0.623	0.631	0.673	0.621	1.000				
9. Interpersonal Influence	0.438	0.523	0.366	0.447	0.420	0.493	0.702	0.583	0.831			
10. Self-Efficacy	0.477	0.403	0.490	0.461	0.680	0.665	0.451	0.566	0.493	0.896		
11. Resource-Facilitating Conditions	0.534	0.394	0.431	0.554	0.668	0.710	0.497	0.580	0.516	0.749	0.928	
12. Technology-Facilitating Conditions	0.283	0.317	0.355	0.401	0.568	0.501	0.372	0.396	0.417	0.533	0.708	0.925
HTMT ratio of correlationsb												
1. Attitudes												
2. Subjective Norms	0.521											
3. Perceived Behavioral Control	0.486	0.490										
4. Usage Intention	0.697	0.524	0.417									
5. Perceived Ease of Use	0.571	0.516	0.545	0.629								
6. Perceived Usefulness	0.742	0.471	0.563	0.702	0.810							
7. Perceived Enjoyment	0.552	0.401	0.358	0.558	0.634	0.724						
8. Perceived Compatibility	0.658	0.441	0.395	0.681	0.662	0.746	0.658					
9. Interpersonal Influence	0.527	0.618	0.431	0.561	0.501	0.626	0.852	0.659				
10. Self-Efficacy	0.540	0.454	0.566	0.543	0.761	0.798	0.510	0.606	0.600			
11. Resource-Facilitating Conditions	0.607	0.450	0.502	0.665	0.761	0.851	0.573	0.627	0.646	0.869		
12. Technology-Facilitating Conditions	0.315	0.370	0.417	0.476	0.642	0.611	0.434	0.425	0.517	0.615	0.835	

Note. The Fornell–Larcker criterion refers to a factor’s average variance extracted, which should be higher than its squared correlation with all other factors in the model. Heterotrait–monotrait ratio significantly smaller than 0.9.

**Table 5:** Results of the 11 Hypothesis Tests in the Structural Equation Model.

Hypothesized Paths		Path Coefficients	t-Value	*p*-Value
H1	Attitudes → Usage Intention	0.481	4.875	<0.001 ***
H2	Subjective Norms → Usage Intention	0.213	2.312	0.021 *
H3	Perceived Behavioral Control → Usage Intention	0.064	0.654	0.513
H4	Perceived Ease of Use → Attitudes	0.021	0.164	0.869
H5	Perceived Usefulness → Attitudes	0.359	2.57	0.010 *
H6	Perceived Enjoyment → Attitudes	0.056	0.447	0.655
H7	Perceived Compatibility → Attitudes	0.341	2.789	0.005 **
H8	Interpersonal Influence → Subjective Norms	0.523	6.431	<0.001 ***
H9	Self-Efficacy → Perceived Behavioral Control	0.380	2.61	0.009 **
H10	Resource-Facilitating Conditions → Perceived Behavioral Control	0.077	0.426	0.670
H11	Technology-Facilitating Conditions → Perceived Behavioral Control	0.098	0.953	0.341

Note. * *p* < 0.05, ** *p* < 0.01, *** *p* < 0.001.

## Data Availability

The data presented in this study are available from the corresponding author upon reasonable request. The data are not publicly available due to privacy restrictions.

## Abbreviations

The following abbreviations are used in this manuscript:

DTPB	Decomposed Theory of Planned Behavior
ISMEP	Interactive Smart Mirror Exercise Program
TPB	Theory of Planned Behavior
TAM	Technology Acceptance Model
PLS-SEM	Partial least-squares structural equation modeling
CR	Composite Reliability
AVE	Average Variance extracted
HTMT	Heterotrait-Monotrait
SRMR	Standardized Root-mean-square Residual
